# How Low-Carbon Pilots Affect Chinese Urban Energy Efficiency: An Explanation from Technological Progress

**DOI:** 10.3390/ijerph192315563

**Published:** 2022-11-23

**Authors:** Jian Song, Jing Wang, Zhe Chen

**Affiliations:** 1 School of Economics, Nanjing Audit University, Nanjing 211815, China; s_ongking@nau.edu.cn; 2 School of Economics, Nanjing University, Nanjing 210023, China; dg20020025@smail.nju.edu.cn

**Keywords:** low-carbon city pilot, energy efficiency, technological progress, difference-in-differences model, energy rebound effect

## Abstract

This study uses the low-carbon city pilot (LCCP) policy as a quasi-natural experiment, combined with the panel data of 281 prefecture-level and above cities in China from 2003 to 2018, and the difference-in-differences model to investigate the specific impact and mechanism of the LCCP on regional energy efficiency. The study showed that LCCP policies can significantly improve regional energy efficiency. The heterogeneity test found that, for cities with larger scales, high pollutant emission intensities, and fewer financial constraints, the implementation of LCCP policies could effectively improve energy efficiency. Based on the mechanical test of the technological progress path, it was concluded that LCCP policies could effectively improve energy efficiency by promoting technological innovation and transformation of enterprises. In the field of technological innovation, implementing LCCP policies helps promote green technological innovation, as well as increasing bias. Furthermore, this study evaluates the emission reduction effects of LCCP policies. The results found that, although LCCP could reduce regional carbon emissions by improving energy efficiency, the impact of energy rebound dramatically reduced the emission reduction effects of LCCP. This study provides empirical evidence and policy enlightenment for China’s accelerating “carbon-peak” and “carbon-neutral” goals.

## 1. Introduction

Global warming has become a common problem that all countries must face in their future economic development. According to the World Meteorological Organization, 2015–2019 comprised the hottest period recorded in the world. In 2019, the average concentration of carbon dioxide in the atmosphere had reached 147% of preindustrial levels. Although this trend significantly declined during the COVID-19 epidemic in 2020, the carbon emission reduction was “short-term and unsustainable,” and the form of global emission reduction is still not optimistic. Therefore, countries around the world have begun to actively respond to global climate governance, accelerate regional low-carbon development and energy structure optimization, and realize the growth of global energy in an efficient, clean, and diversified direction (Wu et al., 2021 [1]; Zeng et al., 2021 [2]). As the world’s largest energy consumer and carbon dioxide emitter, China plays an essential leading role in this process (Cheng et al., 2020 [3]). In September 2020, Chinese president, Xi Jinping announced, at the 75th United Nations General Assembly, that “China will increase its nationally determined contributions by adopting more effective policies and measures, strive to reach CO_2_ emissions peak before 2030 and achieve carbon neutrality before 2060”.

Looking back at China’s environmental governance process, environmental regulation, as a typical means of government intervention, has achieved apparent results in the process. Since 2018, China’s carbon emissions intensity has decreased by 45.8%, compared with 2005, and China met its commitment of “reducing carbon emission intensity by 40–45% compared with 2005 by 2020” early, which was closely related to the implementation of the low-carbon city pilot (LCCP) policy. To ensure that the goal of controlling greenhouse gas emissions by 2020 was achieved, the National Development and Reform Commission of the People’s Republic of China (hereinafter referred to as the National Development and Reform Commission) conducted pilot work in low-carbon provinces and cities in China in 2010. Further, the scope of the pilot project was expanded in 2012 and 2017, by directing each pilot city to develop a low-carbon city development plan based on its resource endowments, technological foundation, and market environment, and to accelerate the promotion of regional green low-carbon city, or province, development.

As the national level attaches great importance to low-carbon pilot projects and the gradual expansion of pilot coverage, LCCP policies are becoming increasingly important to reduce energy consumption and carbon emissions in China. However, to achieve the “carbon-peak” goal, it is necessary to realize the gradual “decoupling” of economic and social development from carbon emission growth. Moreover, improvement in energy efficiency is an effective way of reducing the dependence of economic development on energy consumption and carbon emissions reduction. Therefore, the focus should be on determining whether energy efficiency is affected by LCCP policies. Additionally, the LCCP policy is a comprehensive city-level environmental regulation tool. Ongoing verification of the “Porter hypothesis” shows that improving environmental regulations can effectively force enterprises to innovate technologically (Porter and van der Linde, 1995 [4]; Peuckert, 2014 [5]). Many recent studies have also found that environmental regulations represented by market incentives can stimulate enterprises to engage in green technological innovation activities and to produce more green products (Calel and Dechezlepretre, 2016 [6]; Cui et al., 2018 [7]). However, in addition to technological innovation, technological transformation through new and cleaner production equipment should also be an option of technological advancement for enterprises under environmental regulations (Perino and Requate, 2012 [8]), especially for enterprises with low innovation capabilities. Existing literature focuses on the impact of environmental regulation on energy efficiency and technological innovation (Hu et al., 2020 [9]; Wang et al., 2022 [10]). However, there are few studies on whether the energy utilization efficiency can be achieved from the low-carbon city pilot, and discussion on the impact path especially lacks in-depth and sufficient analysis. Against this background, this study discusses the following three questions: Can China’s LCCP policy enhance regional energy efficiency? Are our technological innovation and transformation the two main technical paths? Considering the energy rebound effect, what is the emission reduction effect of the LCCP policies?

Responding to the limitations of the existing literature, the first main contribution of this study is the fact that, by simulating satellite night light data to examine the energy consumption of cities at the prefecture-level and above, this study examined the implementation effects of LCCP policies from an energy efficiency perspective, and complements the literature on policy evaluation. The second main contribution is that, based on the technological innovation mechanism proposed by the Porter hypothesis, this study further deepens understanding of the technical progress path of enterprises and reveals the impact of technological innovation, green technological innovation, and technological transformation on energy efficiency under the low-carbon pilot city. The third main contribution is that, based on the energy rebound effect, this study discusses the impact of the LCCP policy on regional carbon emissions under the energy efficiency pathway, calculates the value of the energy rebound effect of low-carbon pilot cities and non-low-carbon pilot cities, and evaluates the emission reduction effect of the LCCP policy.

This study is presented as follows. Section 2 presents a brief review of the policy background and literature review. Section 3 describes the empirical model and variables, especially the measurement of energy efficiency. Section 4 presents the empirical results and discussion, including baseline regression results, robustness testing, and heterogeneity analysis. Section 5 analyzes the impact of the LCCP policy on regional energy efficiency under different technological progress paths and evaluates the emission reduction effects of LCCP policies. Section 6 presents the conclusions and policy recommendations.

## 2. Literature Review

Facing the dual challenges of high energy demand and transition to low-carbon emissions required for world economic development, accelerating the promotion of energy efficiency, optimizing energy structure, and achieving regional low-carbon development are hot research topics. Given that environmental regulations are currently an essential means of solving ecological externalities, existing studies focused on the effects of “compliance costs” and “innovation compensation” resulting from environmental regulations. Some scholars believe that environmental regulations reduce enterprises’ energy efficiency by crowding out productive investments and increasing pollution control costs (Geng et al., (2007) [11]; Pan et al., (2019) [12]). However, with the introduction of Porter’s hypothesis, many studies found that the “innovation compensation” effect of environmental regulations could “force” enterprises to undertake technological innovations, enhance environmental quality, and increase enterprise competitiveness (Ambec, 2013 [13]; Milani, 2017 [14]). Mandal (2010) [15] used data from the Indian cement industry to find that environmental regulations strengthened regional energy efficiency. Bi et al., (2014) [16] examined the impact of environmental regulations on the energy efficiency of China’s thermal power generation industry. They found that environmental regulations could effectively improve the energy performance and environmental efficiency of the entire industry. In recent years, relevant scholars have focused on the kinds of environmental regulations. Command-and-control environmental regulations are more stringent and make it easier to decentralize targets through a step-by-step decomposition method. In contrast, market-incentive environmental regulations are more flexible. They incentivize enterprises to innovate through market-based means, such as taxation and subsidies, and promote internalization of the external costs of pollution (Blackman et al., 2018 [17]). Tang et al., (2020) [18] explored the best transition time for environmental regulations and showed that, when the marginal opportunity cost of pollution reduction equals the marginal output of capital investment, the transition from command-and-control to market-incentive environmental regulations enhanced the effect of emission reduction. Guo et al., (2020) [19] found that to promote regional energy efficiency, command-and-control environmental regulations exceeded optimal levels, while market-incentive environmental regulations were still within a reasonable range.

Different types of environmental regulations have heterogeneous effects on energy efficiency and emission reduction effects. As a comprehensive environmental policy, LCCP policy has attracted the attention of many scholars. Although building a complete indicator system to evaluate low-carbon urban development could cover different dimensions and levels (Tan et al., 2017 [20]), given the subjectivity of indicator selection and weighting, it is impossible to measure the implementation effect of the pilot policy from a unified perspective. Many scholars began to conduct relevant research based on LCCP policies. Regarding pollution emissions, Wolff (2014) [21] evaluated the impact of low-carbon zone policies on regional air quality and found that low-emission areas reduced particulate matter emissions by 9%. Its positive effects far surpassed other air quality policies. Gehrsitz (2017) [22] focused on Germany and found that the implementation of low-carbon zone policies could effectively improve the air quality in the region and reduce air pollution emissions in the surrounding areas. Regarding productivity, Cheng et al., (2019) [23] found that LCCP played a significant and ongoing role in promoting urban green GDP and was more effective in large areas, having better infrastructure and technological foundations. Yu and Zhang (2021) [24] investigated the impact of LCCP on carbon emission efficiency using difference-in-differences (DID) and spatial DID methods and found that the carbon emission efficiency of pilot cities showed a significant upward trend. Chen et al., (2021) [25] found that LCCP policies could effectively improve enterprise total-factor productivity, and that technological innovation and resource allocation efficiency were the primary transmission paths.

These studies have analyzed and discussed different perspectives related to environmental regulations and LCCP policies. However, the studies, thus far, have limitations: First, they ignore the critical carrier of energy efficiency in LCCP policies in promoting environmental governance and economic development, and lack a quantitative measurement of prefecture-level city energy consumption. Second, existing research generally focused on the Porter hypothesis, which examines whether environmental regulations can promote technological innovation in enterprises, while ignoring other technical transformation paths that could be selected under environmental regulations. Although many studies showed that LCCP could effectively achieve energy conservation and emission reduction, the existence of energy rebound effects led to uncertainty in the emission reduction effects of LCCP. Therefore, this study examined the specific impact of LCCP on energy efficiency and further evaluated the emission reduction effects, so as to provide a theoretical basis, and policy inspiration, to accelerate China’s energy conservation and emissions reduction.

## 3. Background and Literature Review

### 3.1. China’s LCCP Policy

To actively respond to global climate change and achieve the goal of controlling greenhouse gas emissions by 2020, the National Development and Reform Commission released the “Notice on Carrying out Pilot Work in Low-Carbon Provinces and Regions and Low-Carbon Cities” on 19 July 2010. The five provinces of Guangdong, Liaoning, Hubei, Shaanxi, and Yunnan, and the eight cities of Tianjin, Chongqing, Shenzhen, Xiamen, Hangzhou, Nanchang, Guiyang, and Baoding, were listed as the first batch to apply LCCP. Subsequently, on 26 November 2012, the National Development and Reform Commission issued the “Notice on Launching the Second Batch of National Low-Carbon Provinces and Regions and LCCP Work,” identifying three provinces and cities in Beijing, Shanghai, and Hainan Province, and 26 prefecture-level cities, including Shijiazhuang City, for a new round of low-carbon pilot areas. On 7 January 2017, the National Development and Reform Commission announced the list of the third batch of LCCP. According to the “Notice on Launching the Third Batch of National LCCP Work”, 45 cities (districts and counties), including Wuhai City in the Inner Mongolia Autonomous Region, launched the third batch of low-carbon city pilot projects. From the perspective of geographic distribution, the number of low-carbon pilot cities in the eastern, central, and western regions of China is 52, 18, and 26, respectively, which effectively highlights the comprehensiveness of the implementation of China’s low-carbon pilot policies. Among them, the eastern region has the most significant number of pilot cities, which is closely related to the high energy consumption and pollution emissions caused by the economically active and densely populated eastern region.

As a city-level environmental regulation policy, LCCP has the characteristics of weak constraint, industry specificity, and a combined procedure, compared with other ecological pilot policies. First, the state does not impose restrictions on local governments, but rather encourages pilot cities to promote low-carbon work, based on their conditions. Second, the LCCP policies mainly involve high energy consuming fields, such as industry, construction, energy supply, and waste management. Additionally, the LCCP policies include various types of policy tools, such as command-and-control, market incentives, and voluntary measures, and receive support from various green financial policies, such as industry subsidies and credit concessions. Therefore, based on these characteristics of LCCP, this study comprehensively discusses whether the policies can promote regional energy efficiency. Rather than setting 2010 or 2012 as the time node of LCCP policies, this study comprehensively considers the three batches of LCCP policies. In the following Section 5.2, the time-varying DID model is used to analyze the dynamic effects of the three batches of pilots, respectively. Given that the third node was in 2017, the policy effect of the third batch is specially verified.

### 3.2. Theoretical Mechanism

Accelerating the gradual decoupling of economic development from carbon emissions is currently the key to China’s transformation of traditional economic development models and the promotion of regional green and low-carbon transformation. As typical comprehensive environmental regulations, LCCPs play an important role in the process of the “innovation compensation effect.” That is, LCCPs can effectively “force” enterprises to undertake technological innovation and achieve the coordinated development of environmental protection and enterprise competitiveness. However, for companies with low innovation capabilities, the large investments in R&D investment required result in these companies facing funds shortage and concerns regarding the uncertainty of innovation activities, both of which reduce their motivation to innovate (Manso, 2011 [26]). Therefore, this study focuses on two technological progress paths, namely, technological innovation and technological transformation, and explores the specific impact of LCCP policies on regional energy efficiency.

Technological innovation, as a more direct and proactive form of technological advancement taken by enterprises to respond to the pressure of low-carbon pilot cities, positively influenced the improvement of regional energy efficiency (Herrerias et al., 2016 [27]). However, there are different types of technological innovation, which can be divided into green technological innovation and non-green technological innovation from the environmental perspective. Compared with non-green technological innovations, green technological innovation has the advantages of reducing energy consumption and pollution and improving ecology (Magat, 1987 [28]). Additionally, many studies, such as those by Berrone et al., (2012) [29] and Du and Li (2019) [30], proved that green technological innovation could effectively avoid environmental supervision costs, promote the replacement of fossil energy with clean energy in the production process, and achieve energy efficiency.

A review of relevant documents on LCCP policies found that the tasks of the pilot areas include establishing an accountability system for controlling emissions targets, developing policies to support low-carbon development, and advocating low-carbon green lifestyles. This means that LCCP policies have a certain degree of flexibility and can influence corporate green technological innovation in various ways. Although the target accountability system of LCCP projects increases the environmental governance costs of enterprises and reduces the profits directly realized, it can prompt enterprises to actively reflect on their own deficiencies in green development and continuously reduce environmental costs by improving green manufacturing capabilities (Grossman and Helpman, 2018 [31]). Second, the supporting policies for low-carbon city pilots can ease the enterprises’ financing constraints through various methods, such as fund matching, investment subsidies, and loan interest discounts, and encourage enterprises to invest more resources in green technology research and development (Montmartin and Herrera, 2015 [32]). Additionally, the green lifestyle advocated by the LCCP raised consumers’ awareness of environmental protection and accelerated the shift of consumers’ consumption concepts from traditional consumption to green and sustainable consumption. Consumers pay more attention to corporate behavior (Zhang et al., 2018 [33]). To ensure competitive advantage and market share, companies actively engage in green product innovation activities to meet consumers’ green preferences. Moreover, with the increasing pressure of regional environmental governance and the increasingly urgent transformation of regional low-carbon development, companies gradually increase their choice and preference for green technologies (Buysse and Verbeke, 2003 [34]; Berrone et al., 2012 [29]), which further encourages companies to engage in green technological innovation activities, and improves regional energy efficiency. Based on these analyses, this study proposes Hypothesis 1:

**Hypothesis 1:**  *Low-carbon city pilot policies can improve energy efficiency by encouraging enterprises to engage in technological innovation and green technology innovation, which increases green technology bias.*

Besides technological innovation, investing in new and more environmentally friendly production equipment to achieve technological transformation is an important method for companies to improve energy efficiency, according to current environmental regulations (Perino and Requate, 2012 [8]), especially for companies with low R&D capabilities (Shao et al., 2020 [35]). Considering that traditional production equipment were mostly based on improving productivity as a primary condition, the energy waste and environmental governance costs generated in the process were ignored. Therefore, under the LCCP policy, enterprises gradually use cleaner production equipment to replace traditional equipment, which helps reduce energy consumption and optimizes the energy use structure to achieve energy efficiency. The fact that the production efficiency of traditional production equipment is relatively low, leads to increase in environmental governance costs resulting from LCCP projects, which has a greater impact on traditional production equipment. Compared with newer and cleaner production equipment, the inventory level of traditional production equipment has fallen more sharply. At the same time, the energy consumption intensity of traditional production equipment is significantly higher than that of advanced environmentally friendly new machinery and equipment, and long-term use is not conducive to energy conservation and emission reduction. Given that LCCP projects mainly involve industries, energy supply, waste management and other high energy-consuming fields, it is particularly important to accelerate the pace of capital renewal and deploy newer and cleaner production equipment. Based on the above analysis, this study proposes Hypothesis 2:

**Hypothesis 2:**  *Low-carbon city pilot policies can improve regional energy efficiency by promoting technological transformation of enterprises.*

## 4. Research Design

### 4.1. Measurement Model

The DID method can divide the sample into a “control group” and a “treatment group”, based on the time and object of the policy action, to reasonably evaluate the effect of policy implementation. Therefore, this study used the time-varying DID method to investigate the specific impact of LCCP on regional energy efficiency. The two-way fixed effects model is set as follows:

(1) $$Y_{it}=\alpha_{0}+\alpha_{1}Treated_{i}*Time_{t}+\beta Control_{it}+\gamma_{t}+\mu_{i}++\varepsilon_{it}$$

where i and t represent the prefecture-level city and year, respectively and Y represents energy efficiency. Considering that the LCCP was implemented in three batches, Treated is a dummy variable, indicating whether i is a pilot city, $Time_{t}$ indicates before and after the pilot and Control is a set of control variables. The value γ is the time-fixed effect, μ is the city fixed effect, and ε is the random error term.

### 4.2. Measuring Energy Efficiency in Chinese Cities

Energy efficiency can be divided into input factors based on single-factor energy efficiency (SFEE) and total-factor energy efficiency (TFEE). Compared with *SFEE*, which only includes energy input elements, *TFEE* can fully consider the input of labor, capital, and other aspects in the production process and the substitution relationship between these factors. Färe et al., (1994, 2007) [36,37] proposed the traditional DEA model to measure the efficiency. The directional distance function (DDF) model and the non-radial directional distance function (NDDF) consider the undesired output and are widely used in the measurement of environmental efficiency and energy efficiency (Zhou et al., 2012 [38]; Zhang et al., 2013 [39]; Zhang and Choi, 2013 [40]). The variables and models selected in this study referred to the settings of Zhou et al., (2012) [38], Zhang et al., (2013) [39] and Zhang and Choi (2013) [40], except that K and L were the same and they choose fossil fuel as the input. Therefore, based on the reliability of the regression results, this study used *TFEE* as the primary explanatory variable and *SFEE* for the robustness test. For the *TFEE* evaluation indicators, this study selected labor, capital, and energy as the input elements, gross domestic product (GDP) as the desired output, and carbon dioxide emissions as the undesirable output. Labor input was measured by the number of urban employees. Capital investment was characterized as the capital stock, measured by the perpetual inventory method. The specific formula is $K_{t}=I_{t}+\left(1-\delta_{t}\right)K_{t-1}$ , with 1984 as the base period. The symbol I means fixed-asset investment and the depreciation rate δ is 9.6%.

Energy input was measured by urban energy consumption. However, current data for cities at the prefecture-level in China were missing. Therefore, this study used night light data for simulation measurement. The higher the night light brightness, the more economic activities at night, and the more energy consumption in the region (Amaral et al., 2005 [41]; Chand et al., 2009 [42]). The specific process was as follows. The night light data were obtained from the National Oceanic and Atmospheric Administration, which records the stable night light values of provinces and prefecture-level cities after removing background noise and interference. The night light data was fitted with the energy consumption data of various provinces over the years, released by the National Bureau of Statistics in the “China Energy Statistical Yearbook”, and a significant positive correlation was found between data on night lighting and provincial energy consumption. On this basis, a linear model, without intercept (to avoid the accuracy problem of the downscaling model inversion), was used to decompose the energy consumption data at the province level to the night light data of cities at the prefecture level city level. The energy consumption data of each city were then simulated. Additionally, city-level carbon dioxide emissions at the prefecture-level were obtained by summing the emissions of the counties under their jurisdiction. The specific emission inventory was compiled, based on the latest revision of energy data (2015) from China’s National Bureau of Statistics. By combining input–output data, this study used the non-radial directional distance function (NDDF) to measure the energy efficiency of cities at the prefecture level. The specific method was as follows.

The distance function was divided into the Shepard distance function (SDF) and the direction distance function (DDF). Compared with the SDF model, where expected and undesired outputs change in the same direction, the DDF model could increase expected output and decrease undesired output. However, the expansion or contraction of expected output and undesired output in the DDF model is strictly proportional, and prone to “slack bias.” Thus, Zhang and Choi (2013) [40] improved the DDF model by relaxing the assumptions of proportional changes and proposed the NDDF model.

Assuming there are *n* DMUs in the production system, each DMU consists of input, expected output, and undesired output. The vector form is $x\in R^{m},\;\;y^{d}\in R^{s_{1}},\;\;y^{u}\in R^{s_{2}}$ . This production technology set P can be expressed as:

(2) $$P=\left\{\left(X,Y,C\right):X\;produces\;Y,\;and\;produces\;C\right\}$$

According to Färe et al., (2005) [43], the production technology set P is a closed set, emphasizing that limited input can only produce limited output. Additionally, the production technology set P should meet the two conditions of zero intersection and weak disposability, namely:

If $\left(X,Y,C\right)\in P$ and C=0 , then Y=0
If $\left(X,Y,C\right)\in P$ and 0≤θ≤1 , then $\left(X,\theta Y,\theta C\right)\in P$

where zero intersection means that the discharge of pollutants is inevitably associated with the production process. Weak disposability indicates that while the expected output is reduced, the undesired production is reduced in the same proportion.

The input factor X in this study included capital K , labor L , and energy E . The expected output wass Y and the undesired output was C . Then, the nonparametric production technology set, with constant returns to scale, could be expressed as:

(3) $$P=\{\left(K,L,E,Y,C\right):\overset{T}{\underset{t=1}{\sum }}\overset{N}{\underset{i=1}{\sum }}{µ}_{it}K_{it}\leq K,\;\overset{T}{\underset{t=1}{\sum }}\overset{N}{\underset{i=1}{\sum }}{µ}_{it}L_{it}\leq L,\;\overset{T}{\underset{t=1}{\sum }}\overset{N}{\underset{i=1}{\sum }}{µ}_{it}E_{it}\leq E,\;\sum_{t=1}^{T}\sum_{i=1}^{N}{µ}_{it}Y_{it}\geq Y,\;\sum_{t=1}^{T}\sum_{i=1}^{N}{µ}_{it}C_{it}=C,\;\;\;{µ}_{it}\geq 0\}\;$$

Drawing on the research of Chambers et al., (1996) [44], the DDF in this study was defined as:

(4) $$\vec{D}\left(K,L,E,Y,C;\vec{g}\right)=\sup\left\{\beta :\left(\left(K,L,E,Y,C\right)+\beta \vec{g}\right)\in P\right\}$$

where $\vec{g}=\left(g_{k},g_{L},g_{E},g_{Y},g_{C}\right)$ is the direction vector, and $\beta ={\left(\beta_{k},\beta_{L},\beta_{E},\beta_{Y},\beta_{C}\right)}^{T}$ represents the scaling ratio of input and output factors. The expected output and the undesired output in the DDF model change in the same proportion. To overcome the limitation of the radial hypothesis constraint, we constructed the NDDF:

(5) $$\vec{D_{non}}\left(K,L,E,Y,C;\vec{g}\right)=\sup\left\{\omega^{T}\beta :\left(\left(K,L,E,Y,C\right)+\vec{g}\cdot diag\left(\beta \right)\right)\in P\right\}$$

where $\omega ={\left(\omega_{k},\omega_{L},\omega_{E},\omega_{Y},\omega_{C}\right)}^{T}$ represents the normalized weight vector of the input elements and output. It is worth noting that ω can be given different weights, depending on research needs. This study examined the maximum reduction ratio of energy input and undesired output and the maximum expansion ratio of expected output when the capital and labor input elements remained unchanged, and the weight vector was set as $\omega =\left(0,\;0,\;1/3,\;1/3,\;1/3\right)$ . The corresponding direction vector was $\vec{g}=\left(0,\;0,-E,\;Y,-C\right)$ . Accordingly, we built the following model:

(6) $$\vec{D_{non}}\left(K,L,E,Y,C\right)=\max\left\{\frac{1}{3}\beta_{E}+\frac{1}{3}\beta_{Y}+\frac{1}{3}\beta_{C}\right\}\;s.t.\;\overset{T}{\underset{t=1}{\sum }}\overset{N}{\underset{i=1}{\sum }}{µ}_{it}K_{it}\leq K,\;\;\overset{T}{\underset{t=1}{\sum }}\overset{N}{\underset{i=1}{\sum }}{µ}_{it}L_{it}\leq L,\;\overset{T}{\underset{t=1}{\sum }}\overset{N}{\underset{i=1}{\sum }}{µ}_{it}E_{it}\leq E-\beta_{E}g_{E},\;\;\sum_{t=1}^{T}\sum_{i=1}^{N}{µ}_{it}Y_{it}\geq Y+\beta_{Y}g_{Y},\;\sum_{t=1}^{T}\sum_{i=1}^{N}{µ}_{it}C_{it}=C-\beta_{C}g_{C},\;{µ}_{it}\geq 0,\;\beta_{E},\beta_{Y},\beta_{W},\beta_{S}\geq 0$$

By solving Equation (6), the optimal solution $\beta^{*}={\left(\beta_{E}^{*},\beta_{Y}^{*},\beta_{C}^{*}\right)}^{T}$ could be obtained, and the energy efficiency index could be constructed according to the optimal solution; that is, the energy performance (the ratio of the target energy intensity to the actual energy intensity) and the carbon dioxide emission performance (the ratio of the target carbon dioxide emission intensity to the actual carbon dioxide emission intensity) were used for weighting, as follows:

(7) $$TFEE_{it}=\frac{1}{2}\left(\frac{\left(E_{it}-\beta_{E}^{*}\times E_{it}\right)/\left(Y_{it}-\beta_{Y}^{*}\times Y_{it}\right)}{E_{it}/Y_{it}}\right)+\frac{1}{2}\left(\frac{\left(C_{it}-\beta_{C}^{*}\times C_{it}\right)/\left(Y_{it}-\beta_{Y}^{*}\times Y_{it}\right)}{C_{it}/Y_{it}}\right)=\frac{\frac{1}{2}\left(1-\beta_{E}^{*}\right)+\frac{1}{2}\left(1-\beta_{C}^{*}\right)}{1+\beta_{Y}^{*}}$$

### 4.3. Other Variables and Data Sources

#### 4.3.1. Mediating Variable

The mediating variables in this study were the several paths of technological progress, mainly including the two forms: technological transformation and technological innovation. The green technology innovation path and green technology bias are further discussed under the technical innovation path.

- Technological transformation (Tech_trans)

Technological transformation is apparent in the use of new, environmentally friendly machinery and equipment in the production process. This study measured the logarithm of total investment in fixed assets of prefecture-level cities, based on the availability and completeness of the data. The larger the value, the faster the pace of capital renewal in the region and the higher the level of technological transformation.

- Technological innovation (Tech_Inno)

Considering the effect of patents on enterprise performance during the application process, this study used the total number of patent authorizations in prefecture-level cities to characterize technological innovation, with a unit of ten thousand. The larger the value, the higher the regional technological innovation level.

- Green technology innovation (Green_Inno)

The number of green patents applied for each year by cities at the prefecture level was matched against the “International Patent Green Classification List”, announced by the World Intellectual Property Organization in 2010. In this study, alternative energy production, waste management, and energy conservation were the specific items of green patents. This study adopted the measurement of the total number of green patent authorizations, and the larger the value, the higher the level of green technology innovation.

- Green technology bias (Green_Bias)

This study used the proportion of the number of green patent applications in the total number of patent applications in prefecture-level cities to measure the green technology bias. The larger the value, the more likely the region was to engage in green technology research and development.

#### 4.3.2. Control Variables

Regarding the control variables, this study mainly included the following. Per capita GDP ( Pgdp ), obtained by dividing the gross regional product by the total population at the end of the year, was used to represent the level of urban economic development. Population density ( Pdensity ), calculated by dividing the population by the area of the administrative area, was used to reflect the differentiated impact of the scale of urban human activities. Industrial structure ( Indstru ), measured by the proportion of the added value of the secondary industry in the regional GDP, was used to reveal the characteristics of a city’s industrial structure. Pollutant emissions ( Pollu ), measured by the logarithm SO_2_ emissions, represented the level of urban pollution emissions. The energy price ( Price ), was calculated by selecting the purchase index of the fuel and power industrial products of each province or city in China with reference base year 2000. Environmental regulation ( Regulation ) represented the average costs of pollutant discharge, that is, the total amount of the pollution charges over the number of enterprises paying the pollutant charges. Environmental regulation included command-based environmental regulation (Lanoie et al., 2008 [45]; Rubashkina et al., 2015 [46]; Chen et al., 2021 [47]) and market-based environmental regulation, represented by the emissions trading scheme (Carlson et al., 2000 [48]; Franco and Marin, 2017 [49]). This study added the former regulation to assess the net effect of the low-carbon pilot. Fiscal dependence ( Strpub ) adopted the proportion of local general public budget revenue to GDP, indicating that the higher the dependence, the more energy consumption could reduce energy efficiency. Foreign direct investment ( Fdi ) was the logarithm of the actual use of foreign capital after the exchange rate for the current year. The descriptive statistics of the main variables in this study are shown in Table 1.

#### 4.3.3. Research Data

Considering the data availability and consistency, the research sample consisted of the panel data of 281 prefecture-level, and above, cities in China, from 2003 to 2018. The data on patent and green patent applications were obtained from the official website of the State Intellectual Property Office. Other data were acquired from the “China City Statistical Yearbook”, “China Energy Statistical Yearbook”, and “China Industrial Statistical Yearbook” Concurrently, this study used the GDP deflator to deflate all currency volumes into comparable prices, with 2003 as the base period.

## 5. Empirical Results Analysis

### 5.1. Baseline Results

Based on the baseline model constructed earlier, this section examines the impact of LCCP on energy efficiency. The specific regression results are shown in Table 2, where columns (1) and (4) are the pooled ordinary least square (POLS) models, columns (2) and (5) are year-fixed effects models, and columns (3) and (6) are the two-way fixed-effects model of year and city. Using the Hausman test, this study finally selected the two-way fixed-effects model as the main result of the baseline regression. From the regression results in columns (3) and (6), regardless of whether control variables were added, the regression coefficients of Treated∗Time to *TFEE* were all positive at the 1% significance level, indicating that implementing LCCP policies significantly promoted regional energy efficiency.

In terms of control variables, Pgdp and Pdensity had a significantly positive impact on energy efficiency, indicating that improved regional economic development and increased scale of human activities facilitated the improvement of energy efficiency. Indstru had a significantly negative impact on energy efficiency; that is, a higher proportion of secondary industry was less conducive to improving urban energy efficiency. This was closely related to the fact that high-carbon energy, such as coal and petroleum, dominate the energy structure of secondary industry. Additionally, increase in Pollu also significantly inhibited the improvement of energy efficiency. The results of Price and Strpub were significantly negative, indicating that rising energy prices and over-reliance on local fiscal assistance reduced urban energy efficiency. This study found no significant effect of Regulation and Fdi .

### 5.2. Parallel Trend Test

The experimental and control groups conformed to the parallel trend assumption, which is a prerequisite for the consistency of DID estimation results. To further test the parallel trend assumption and the dynamic processing effects of LCCP over time, this study drew on the event research method of Jacobson et al., (1993) [50], to test the differences in the implementation of LCCP at different times. The specific model was set as follows:

(8) $$Y_{it}=\alpha_{0}+\sum_{m\geq -6,m\neq -1}^{m=6}\rho_{m}D_{i}^{m}+\beta Control_{it}+\gamma_{t}+\mu_{i}+\varepsilon_{it}$$

where $D_{it}^{m}$ is a dummy variable, indicating whether the city i in year t is in the m th year before or after implementing the LCCP. The three low-carbon pilots in this sample were time-varying experiments, which included pre-14 years to post-8 years. Referring to Beck and Levkov (2010) [51], this study adopted the tail-shrinking strategy to set more than 6 years as the 6th year. If m=−6 , this indicated the 6th year before the pilot city implemented the policy, and the rest m=−5,−4,−3,−2, 0, 1, 2, 3 , 4, 5, 6 and so on. To capture the difference in results between the treatment and control groups before and after the implementation of the LCCP, Equation (8) omitted the dummy variable when m=−1 .

Table 3 shows the test results of the parallel trend test of LCCP on energy efficiency. Before implementing the LCCP policy, regardless of the addition of the control variables, there was no difference in energy efficiency between the treatment and control groups. Thus, the parallel trend assumption was satisfied. After implementing the LCCP policy, the energy efficiency of the treatment and control groups of cities began to diverge. Compared to the control group cities, the energy efficiency of the treatment group cities was significantly positive after implementing the policy, further verifying the effectiveness of the LCCP policy in improving regional energy efficiency.

**Figure 1:**
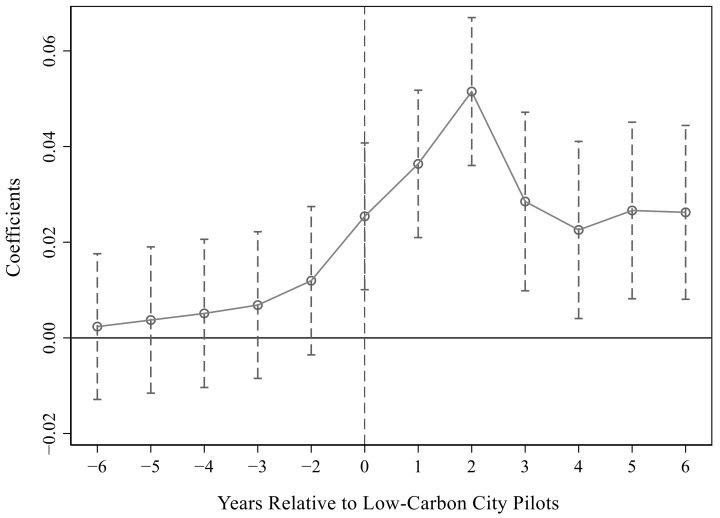
Parallel trend test in the first column of Table 3.

**Figure 2:**
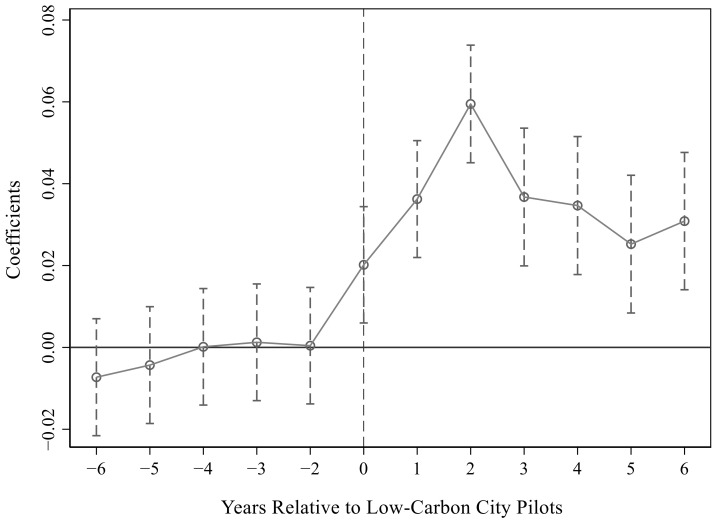
Parallel trend test in the second column of Table 3.

**Figure 3:**
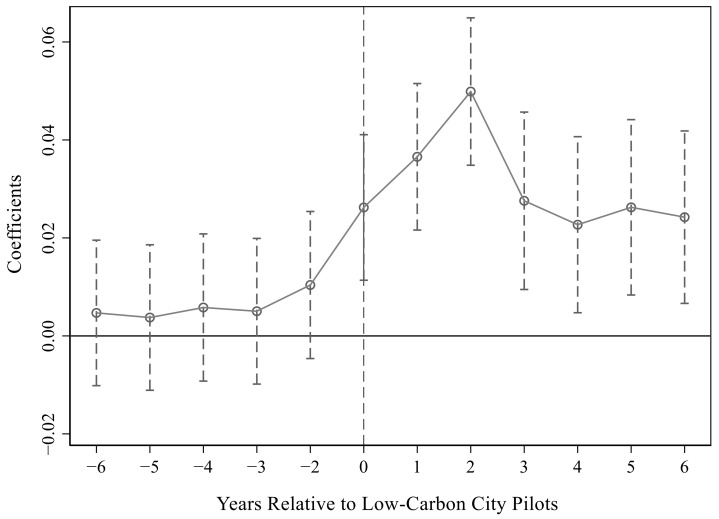
Parallel trend test in the third column of Table 3.

For Table 3, we drew the $\rho_{m}$ estimation results under the 95% confidence interval, which are shown in Figure 1, Figure 2 and Figure 3. Obviously, the parameter values were all about zero before the pilot and were not significant. After the pilot, the parameters gradually became larger and significant, especially in the first two years after the pilot. This showed that there was no significant difference between the treatment group and the control group before the implementation of the pilot policy, which satisfied the parallel trend assumption. That is, it verified that the urban low-carbon pilot significantly improved energy efficiency.

### 5.3. Robustness Test

To verify the robustness of the reference regression results, this study used many methods for robustness analysis, including replacing the explained variables, excluding other policy influences, changing the estimation method, and testing the dynamic effect.

#### 5.3.1. Replace the Explained Variable

This study used *SFEE* as a replacement variable for *TFEE*. *SFEE* measures by using the ratio of regional GDP to energy consumption. The larger the value, the higher the energy efficiency. The specific regression results are shown in column (1) of Table 4. The regression coefficient of the LCCP policy on *SFEE* was positive at a significance level of 1%, and the coefficient signs and significance of control variables did not change much, which was consistent with the baseline regression results.

#### 5.3.2. Exclude Other Policy Influences

The “Resource-based City Sustainable Development Plan”, promulgated by the State Council, and the “National Old Industrial Base Adjustment and Transformation Plan (2013–2020)”, approved by the State Council in 2013, both encourage energy efficiency improvement from different perspectives. Therefore, to accurately assess the policy effects of LCCP, this study excluded the data from 2013 to 2018 to avoid interference from other policies on the accuracy of the regression results. The specific regression results are shown in column (2) of Table 4. After excluding the influence of other policies, the regression coefficient of the LCCP policy was still significantly positive at 1%, which effectively guaranteed the robustness of the regression results in this study.

#### 5.3.3. PSM–DID

Due to the possible selectivity bias of the DID model, there is no guarantee that the treatment and control groups had the same individual characteristics before the policy was implemented. This study further used the PSM–DID model for estimation. Specifically, this study first used the propensity score matching (PSM) method to use observable variables to match each city in the treatment and control groups. The matched treatment and control groups had the same probability distribution. The maximum distance allowed between the experimental group and the matching group was 0.05, and neighbor matching was performed according to ratio 1:3. Subsequently, the difference method was used to perform a regression analysis on the matched results. The specific results are shown in column (3) of Table 4. The impact of LCCP on energy efficiency was still significantly positive, indicating that the conclusions drawn in this study were robust.

#### 5.3.4. Dynamic Window Test

The regression results of this study showed that the LCCP had effectively promoted energy efficiency improvement. However, it failed to reflect differences in the promotion effect at different periods before and after the policy was promulgated. Therefore, this paper further tested by changing the time window before and after implementing the LCCP policy. Considering that the list of LCCP participants was released in 2010, 2012 and 2017, this study tested the three pilots in the different dynamic periods, respectively. Treated2010, Treated2012 and Treated2017 were the pilot cities in the corresponding years. Table 5 shows the specific regression results. Regardless of whether 2010, 2012 or 2017 was the policy time node, change in the time window did not affect the promotion of LCCP policies on energy efficiency, effectively ensuring the reliability of the regression results. Moreover, as the time window increased, the regression coefficients of LCCP were stable and the level of significance was significantly robust, effectively highlighting the effectiveness and sustainability of policy implementation.

The above is a dynamic window test for the three pilots, respectively. The premise was that all the pilots satisfy the parallel trend assumption. In order to avoid the mutual influence of pilots in different years, this study excluded the other two pilot cities for separate testing, and mainly focused on the first two periods after the pilot. In this study, the sample period of the first pilot was shortened to before 2013, and the second pilot to before 2015. The third pilot started in January 2017, and in order to be consistent with the previous two, the policy year was set to 2016. Of course, we also verified that setting it as 2017 did not change the result, and was just one less year. Table 6 shows that the coefficients of the three pilots were close to 0 and insignificant before the pilots, and the coefficients became larger and significant after the pilots. Therefore, it was verified that the three pilots met the parallel trend assumption; that is, the results of the dynamic analysis were robust.

### 5.4. Heterogeneity Analysis

Considering that the economic levels and development environments of cities show significant imbalances, the impact of LCCP on energy efficiency might differ regionally. Therefore, this study examined whether the effect of LCCP on energy efficiency was heterogeneous in different regions, from the perspectives of city size, environmental regulation intensity, and financing constraint pressure.

#### 5.4.1. City Size

Cities of different sizes show obvious differences in economic development, innovation capabilities, and resource allocation. In general, the larger the city, the more likely it can address environmental governance issues in a timely and accurate manner and more effectively conserve energy and reduce emissions. Therefore, in accordance with the “Notice on Adjusting the Standards for Urban Size Classification”, issued by the State Council, this study divided cities into small- and medium-sized cities (with a population of less than 1 million) and large cities (with a population of 1 million or more). Large cities were divided into three types: Type II large cities (populations greater than, or equal to, 1 million and less than 3 million), Type I large cities (population greater than, or equal to, 3 million and less than 5 million), and extra-large and above cities (population greater than, or equal to, 5 million).

Table 7 shows the heterogeneous impacts of the LCCP on regional energy efficiency at different city scales. The regression coefficients of the LCCP in small- and medium-sized cities (SMCs) were significantly negative, which was not conducive to improving regional energy efficiency. In contrast, the LCCP can effectively promote the advancement of regional energy efficiency in large cities (LCs). It was further found that the regression coefficients of the LCCP in type II large cities (II-LCs) were not significant, while type I large cities (I-LCs) and the extra-large and above cities (ELCs) were both positive, with at least 5% significance levels. Therefore, the LCCP showed substantial differences in the energy efficiency of cities of different sizes. When the city scale reached a certain level, the implementation of LCCP policies could effectively improve regional energy efficiency. Moreover, the larger the city, the more pronounced the improvement.

#### 5.4.2. Pollutant Emission Intensity

Environmental regulation is an important policy tool for governments to intervene in environmental governance. In areas with a high level of pollutant emissions, implementing LCCP policies was more conducive to promoting enterprises undertaking technological innovation, achieving efficiency improvements, energy-saving and reducing emissions. In contrast, companies in areas with low levels of pollutant emission might prefer to implement end-of-pipe governance when confronted with LCCP policies. The pollutant emission intensity indicator, Environmental regulation intensity index, was constructed. First, industrial wastewater, industrial SO_2_, and industrial smoke and dust emissions were selected as the primary pollutants, and the pollutant emissions per unit output value of each city calculated. Second, the pollutant emission value per unit output value of each city was linearly standardized according to the value range of [0, 1]. Finally, the standardized value of each pollutant was weighted and averaged with equal weights, and the intensity of pollutant emission in each city obtained.) This study divided the cities into high (HPEI) and low (LPEI) groups, based on the average value of pollutant emission intensity during the inspection period. The study examined the heterogeneous impact of LCCP on regional energy efficiency at different intensities of pollutant emissions. The specific regression results are shown in columns (1) and (2) of Table 7. The results showed that LCCP could effectively promote energy efficiency improvement in areas with high and low environmental regulatory intensity. However, regions with high pollutant emissions had greater positive policy effects than those with low emissions. Therefore, the promotion of regional energy efficiency through LCCP policies should be supported by pollutant emission intensity.

#### 5.4.3. Financing Constraint

Some companies may face financing constraints and lack the motivation for low-carbon transformation through innovation by the implementation of the LCCP policies. Therefore, this study used the average value of loan balances from financial institutions during the sample period to divide cities into two groups, high (HFC) and low (LFC), to study the differential impact of LCCP on energy efficiency under different financing constraints. HFC indicated that the urban enterprises concerned obtained more funds and faced less financing constraints, while LFC indicated that they obtained less funds and faced larger financing constraints. The results are shown in columns (3) to (4) of Table 8. The regression coefficient of LCCP policies was not significant in areas with high financing constraints pressure, as seen in column (3). Nevertheless, it was significantly positive in regions with low financing constraints pressure, as seen in column (4). Therefore, a relatively complete credit policy, especially a green financial policy, could ease financial pressure for enterprises seeking to technologically innovate, which would be conducive to improving regional energy efficiency.

## 6. Mechanism Test and Emission Reduction Effect Analysis

### 6.1. Mechanism Inspection Based on the Path of Technological Progress

This study built a model of mediating effects to test the specific impact of LCCP on energy efficiency through technological transformation, technological innovation, and green technology innovation (Baron and Kenny, 1986 [52]). The model was constructed as follows:

(9) $$M_{it}=\theta_{0}+\theta_{1}Treated_{i}*Time_{t}+\rho Control_{it}+\gamma_{t}+\mu_{i}+\varepsilon_{it}$$

(10) $$Y_{it}=\omega_{0}+\omega_{1}Treated_{i}*Time_{t}+\omega_{2}M_{it}+\varphi Control_{it}+\gamma_{t}+\mu_{i}+\varepsilon_{it}$$

Combining (1), (9), and (10) gave rise to the mediating effect model of this study. M represents an intermediate variable, including technological transformation, technological innovation, green technological innovation, and green technological bias indicators. The value $\alpha_{1}$ represents the total effect of LCCP policies on regional energy efficiency, $\theta_{1}\omega_{2}$ is the mediating effect transmitted through the mediating variable M , and $\omega_{1}$ is the direct effect. If the regression coefficient $\alpha_{1}$ in Equation (1) was significant, there was a mediation effect argument. It was then necessary to check $\theta_{1}$ in Equation (9) and the regression coefficient $\omega_{2}$ in Equation (10), and if both were significant, then there was a mediation effect. Finally, it was necessary to check the regression coefficient $\omega_{1}$ in Equation (10). If it was not substantial, it was a complete mediation effect. If it was significant, it was a partial mediation effect, and the proportion of the mediation effect in the total effect was $\theta_{1}\omega_{2}/\alpha_{1}$ .

#### 6.1.1. Technological Transformation and Technological Innovation

Table 9 shows the mediation effect test results, based on technological transformation and innovation. As can be seen from columns (1) and (3), the increase in the number of fixed-asset investments and patents had a significantly positive impact on regional energy efficiency, which meant that investing in new and clean equipment or increasing R&D investment was conducive to improving energy efficiency. It could be concluded from columns (2) and (4) that the regression coefficients of the LCCP policies were positive at the 1% significance level, indicating that LCCP policies could effectively encourage enterprises to engage in technological transformation and innovation. Therefore, combined with the baseline regression results in Table 2, it was concluded that, in the LCCP policies to promote regional energy efficiency, both technological transformation and innovation were effective mediating paths. It is worth noting that the mediation effect of technological transformation accounted for 6.41% of the total effect, while that of technological innovation accounted for 43.36% of the total effect. This showed that enterprises were more likely to achieve energy efficiency improvements through technological innovation under the LCCP policy, rather than through technological transformation.

#### 6.1.2. Green Technology Innovation and Green Technology Bias

These results verified that technological innovation is a critical path for the current LCCP policies to promote regional energy efficiency. Furthermore, this study started from green technology and examined the mediating effect of green technology innovation and bias. The specific regression results are shown in Table 10. Columns (2) and (4) show that the regression coefficients of the LCCP are all positive at the 1% significance level, indicating that the LCCP policies promoted not only enterprises engaging in green technological innovation, but also technological progress in a green direction. Columns (1) and (3) show a significantly positive impact of green technology innovation and bias on regional energy efficiency, effectively highlighting the positive role of green development in improving regional energy efficiency. The mediation effect of green technology innovation accounted for 31.58% of the total effect, while that of green technology bias accounted for 8.14% of the total effect. Therefore, combined with the baseline regression results in Table 2, it was shown that LCCP could effectively improve energy efficiency by encouraging enterprises to engage in green technology innovation and fostering enterprises’ green technology bias.

#### 6.1.3. Parallel Trend Test of Mediating Variables

In this study, the time-varying DID method was used to design the mediating effect. In order to ensure the causal relationship of the mediating variables, the parallel trend assumption should be satisfied. In this section, we used formula (8) to replace the explanatory variable with the mediator variable. Table 11 reports the test results of the mediator variables. Obviously, the coefficients of Tech_trans , Tech_Inno , Green_Inno and Green_Bias were not significant before the pilot. Tech_trans was significant from the current year of the pilot (*D*^0^), Tech_Inno was significant from the post-second year (*D*^2^), Green_Inno was significant from the current year (*D*^0^), and Green_Bias was significant from the post-first year (*D*^1^). Thus, it was verified that the above mechanism variables satisfied the parallel trend assumption, which ensured the reliability of the mechanism results.

### 6.2. Emission Reduction Effects of LCCP

#### 6.2.1. The Results of Emission Reduction Effects

China faces the challenges of accelerating regional low-carbon transformation and achieving carbon emission reduction targets. This study shows that the LCCP policies play a significant role in promoting regional energy efficiency. Moreover, existing research generally assumes that technological progress and increased efficiency are the main driving factors behind the current CO_2_ emissions reduction (Ang, 2009 [53]; Okushima and Tamura, 2010 [54]). This means that improvement in energy efficiency plays a vital role in reducing carbon emissions through LCCP policies. However, in fact, energy efficiency improvement has a dual impact on carbon emissions. While reducing carbon emissions, the input of energy elements is also increased, due to the energy rebound effect, resulting in increase in carbon emissions, which partially or fully offsets the reduction in carbon emissions (Sorrell, 2008 [55]; Zhang et al., 2017 [56]).

Specifically, on the one hand, the improvement of energy efficiency can lower the price of effective energy services, which affects the income effect of energy consumers and the substitution effect of energy and other factors of production, thereby increasing the demand for energy consumption. On the other hand, the improvement of energy efficiency promotes output growth, leading to more energy input (Chen et al., 2021 [57]). Therefore, the energy rebound effect creates uncertainty regarding the emission reduction effects of LCCP.

Based on this analysis, this study first examined the impact of LCCP on regional carbon emissions under the energy efficiency path. The model was constructed as follows:

(11) $$CO2_{it}=\theta_{0}+\theta_{1}Treated_{i}*Time_{t}+\rho Control_{it}+\gamma_{t}+\mu_{i}+\varepsilon_{it}$$

(12) $$CO2_{it}=\omega_{0}+\omega_{1}Treated_{i}*Time_{t}+\omega_{2}TFEE_{it}+\varphi Control_{it}+\gamma_{t}+\mu_{i}+\varepsilon_{it}$$

where combining (1), (11), and (12) gave rise to the mediating effect model of this study. CO_2_ is the explained variable, indicating the degree of urban carbon emissions. *TFEE* is the mediating variable. The specific regression results are shown in Table 12. Columns (1) and (3) show that the impact of LCCP on regional CO_2_ emissions was negative at a 1% significancy level, which effectively highlighted the effectiveness of LCCP policies in energy conservation and emission reduction. Moreover, the impact of energy efficiency on regional CO_2_ was also significantly negative, indicating that improved energy efficiency could drastically reduce carbon emissions. The results in column (2) were consistent with the baseline regression results. Therefore, the LCCP policies could effectively achieve emission reduction effects by improving regional energy efficiency. However, it is worth noting that the mediating effect of energy efficiency accounted for only 9.44% of the total effect. This showed that under the energy efficiency mediating path, LCCP policies played a small role in reducing carbon emissions. This study speculated that the presence of energy rebound effects might result in LCCP failing to achieve the expected emission reduction effects.

#### 6.2.2. Parallel Trend Test for Carbon Reduction

This part of the study analyzed the emission reduction effect of LCCP, and the explanatory variable was replaced by CO_2_, which also needed to meet the parallel trend assumption. Table 13 reports the parallel trend test for CO_2_ emission reduction, in which a two-way fixed-effects model was used in column (1) and control variables were added in column (2). The results of both columns showed that the coefficients before the pilot were about zero and not significant, and they were significant from the pilot year. Thus, it was verified that the carbon reduction effect of LCCP satisfied the parallel conditions.

**Figure 4:**
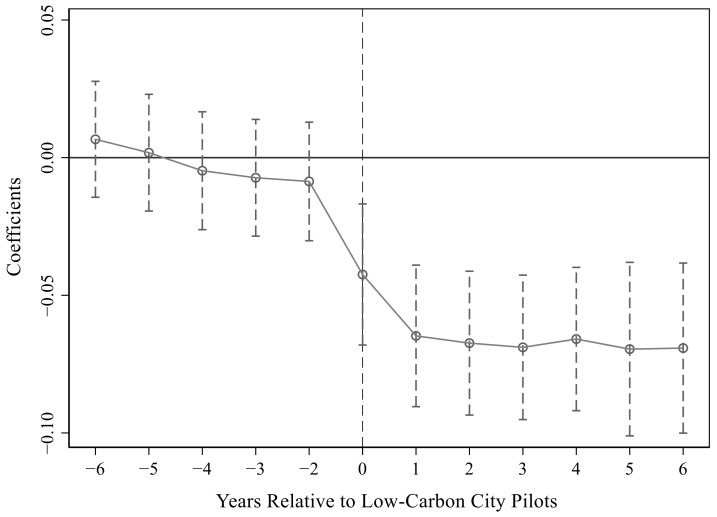
Parallel trend test in the first column of Table 12.

**Figure 5:**
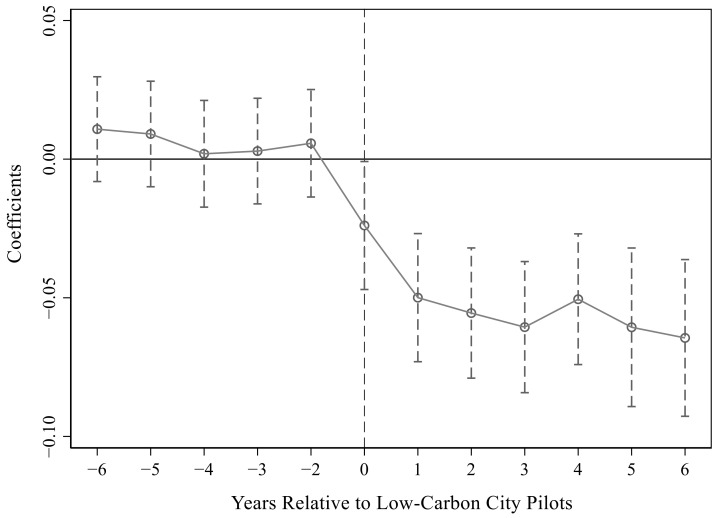
Parallel trend test in the second column of Table 12.

Figure 4 and Figure 5, in combination with the results in Table 13, visually show the dynamic parallel trend effect of carbon reduction. Figure 4 is the result for column (1) in Table 12, and Figure 5 is the result for column (2). Therefore, under the premise of satisfying the parallel trend assumption, this study verified that LCCP significantly reduced the carbon emission reduction effect, indicating that there was a causal relationship between the two.

#### 6.2.3. Energy Rebound Effect

Furthermore, to verify conjecture, this study estimated the value of the city-level energy rebound effect. The rebound effect of energy is reflected in reduction in energy service prices and increase in output. However, China’s energy price has gone through three stages of unified pricing, dual-track pricing, and market-oriented reform, leading to the incomparability of energy prices at different stages (Shao et al., 2014 [58]). Additionally, there is a serious lack of city-level data about energy prices. Therefore, this study measured the energy rebound effect of each city from the perspective of increased output resulting from improved energy efficiency (Shao et al., 2019 [59]). The specific formula was as follows:

(13) $$ERE=-\frac{\rho_{t}\left(Y_{t}-Y_{t-1}\right)I_{t}}{Y_{t}\left(EI_{t}-EI_{t-1}\right)}$$

where $Y_{t}$ and $Y_{t-1}$ represent the economic output levels in period t and t−1, respectively, and $EI_{t}$ and $EI_{t-1}$ represent energy intensity in period t and t−1, respectively. On the one hand, the improvement of energy efficiency in period t reduces energy intensity, which. in turn. reduces the energy consumption of $Y_{t}\left(EI_{t}-EI_{t-1}\right)$ . On the other hand, the improvement of energy efficiency in period t promotes output growth $\rho_{t}\left(Y_{t}-Y_{t-1}\right)$ , thereby increasing the energy consumption of $\rho_{t}\left(Y_{t}-Y_{t-1}\right)EI_{t}$ . Therefore, the absolute value of the ratio of the energy consumption of the latter to the former is the formula for the energy rebound effect at the macro-level. It is worth noting that $\rho_{t}$ is the contribution rate of energy efficiency improvement to output growth, and the formula is $\rho_{t}=\frac{\dot{TFEE_{t}/}TFEE_{t-1}}{\dot{Y_{t}}/Y_{t-1}}$ .

According to formula (13), this study calculated the energy rebound effect of all cities, pilot cities, and non-pilot cities. The specific results are shown in Table 14. Overall, China’s energy rebound effect value, from 2004 to 2007, was about 85.18%, implying that 85.18% of the emission reduction effect resulting from improved energy efficiency was reduced. The LCCP in 2010 and 2012 were used as the time nodes to assess the energy rebound effect. The pilot cities promulgated in 2012 and 2017 were eliminated with 2010 as the time node, and the pilot cities promulgated in 2017 were eliminated with 2012 as the time node. Comparing pilot and non-pilot cities, whether 2010 or 2012 was the time node, it was found that after the time node, the energy rebound effect of the pilot cities over time was greater than that of the nonpilot cities. However, before the time node, the relationship between the two showed unstable wave dynamics. Additionally, the average energy rebound effect of non-pilot cities was 82.5%, while that of pilot cities reached more than 90%. Moreover, the number of pilot cities with 2012 as the time node included the low-carbon cities promulgated in 2010 and 2012, resulting in a relatively larger number of pilot cities and an obvious energy rebound effect brought about by the LCCP policies. Therefore, although implementing LCCP policies improved energy efficiency, it resulted in increased output and lower energy service prices, thus, continuously increasing the input of energy factors, which significantly reduced the emission reduction effect of LCCP.

## 7. Conclusions and Policy Recommendations

Based on the panel data of 281 prefecture-level and above cities in China, from 2003 to 2018, this study used the DID model to examine, in depth, the impact of LCCP policies on regional energy efficiency and the specific mechanisms involved. The main conclusions are as follows. First, LCCP significantly promoted the improvement of regional energy efficiency and passed a series of robustness analyses, such as replacing the explained variables, eliminating other policy influences, PSM–DID testing, and dynamic time window testing, to ensure the reliability of the regression results. Second, there were obvious differences in the impact of LCCP on the energy efficiency of different regions. For regions with large cities, high pollutant emission intensity, and less financing constraints, implementing LCCP policies could significantly improve regional energy efficiency. Third, technological innovation and transformation were both effective mediating paths for LCCP policies to promote regional energy efficiency. However, the mediating effect of technological innovation accounted for a more significant proportion of the total effect. Regarding the path of technological innovation, the implementation of the LCCP policies encouraged enterprises to engage in green technological innovation, cultivated enterprises’ green R&D bias, and, thereby, achieved energy efficiency improvement. Fourth, LCCP policies significantly promoted the reduction of regional carbon emissions by improving energy efficiency. However, the mediating effect of energy efficiency accounted for only 3.53% of the total effect, which was related to energy rebound effects. Regardless of whether 2010 or 2012 was the policy time node, the average energy rebound effect of the pilot cities was significantly larger than that of the non-pilot cities. After implementing the LCCP policy, the energy rebound effect of the pilot cities over time was larger than that of the nonpilot cities.

Based on the above conclusions, the policy recommendations in this study are as follows: (1) Continue to promote LCCP policies to achieve urban green and low-carbon transformation. The LCCP policy can effectively promote the improvement of regional energy efficiency. Therefore, to accelerate the gradual decoupling of economic development and carbon emissions, and early achievement of the carbon-peak and carbon-neutral goals, the LCCP work should be further intensified. Although the LCCP promotes the continuous expansion of the coverage of LCCP, regular reviews of the pilot work, active promotion of the development experience of successful LCCP areas, and accelerating the promotion of comprehensive low-carbon city development are weakly binding. To avoid image projects and conceptual hype, the government should play an influential leadership and supervisory role in implementing the LCCP and actively promoting regional pilot work. (2) Companies should choose technological advancements based on their comparative advantages, to respond appropriately to environmental regulations and policies. Technological innovation and transformation are both effective mediating paths for LCCP policies to promote regional energy efficiency. Considering that different companies have apparent differences in resource endowments, technological foundations, and innovation capabilities, companies in each region should choose a technical advancement method that suits their own development, based on their comparative advantages. For example, under the implementation of LCCP policies, enterprises with relatively high innovation capabilities should actively engage in technological innovation, particularly green technology innovation, by accelerating the large-scale application of energy-saving low-carbon technologies and eliminating low-end outdated production capacity to achieve optimal resource allocation. Companies with low R&D capabilities should adopt a gradual development approach, shifting from technological transformation to technological innovation, and should avoid excessive pursuits of R&D investment that impedes the sustainable development of the company. (3) Promoting the clean transformation of energy structure and actively encouraging the development of new energy technologies are necessary. The energy rebound effect has dramatically reduced the emission reduction effect of LCCP policies. Therefore, in advancing the process of energy conservation and emission reduction, we must focus on improving energy use efficiency and accelerate the optimization and adjustment of energy structures. Although enterprises must gradually realize the transition from high-carbon energy and fossil energy to low-carbon energy and renewable energy, especially for industrial sectors characterized by “high energy consumption” and “high emission,” to continuously promote the development of clean energy, the government must continue to increase R&D and subsidies in the field of new energy technologies, actively assist relevant technology companies in expanding market channels, shorten the process of the industrialization of results, and institutionally ensure efficient energy use and the effective diffusion of new technologies.

Finally, this study has certain limitations. Owing to the limitations of micro-level energy data, this study only analyzed the relevant research on LCCP from the level of cities at the prefecture level. Second, the heterogeneous analysis of financing constraint pressure also had data limitations and lacked more intuitive green finance data. Additionally, the presence of the energy rebound effect prevented the LCCP from achieving the expected emission reduction effects. How to overcome the energy rebound effect remains to be studied.

## Figures and Tables

**Table 1:** Descriptive statistics.

Variables	Obs	Mean	Std.Dev.	Min	Max
TFEE	4495	0.1985	0.1301	0.0086	1.3532
SFEE	4495	1.1656	0.9281	0.0160	6.0912
Pgdp	4495	8.5036	0.7538	6.6034	10.1535
Pdensity	4495	5.7150	0.8819	2.3598	7.7003
Indstru	4495	0.4817	0.1126	0.1710	0.8224
Pollu	4495	10.3880	1.1480	5.6821	12.7097
Price	4495	4.8652	0.1843	4.5476	5.3684
Regulation	4495	1.6296	0.6738	0.5005	3.4798
Strpub	4495	6.4214	0.4059	5.3837	7.5115
Fdi	4495	7.6700	1.9796	1.3370	12.1068
Tech_trans	4408	1.9878	0.9851	0.2440	4.4294
Tech_Inno	4479	0.2063	0.3733	0.0006	1.4752
Green_Inno	4417	0.0130	0.0234	0.0001	0.0928
Green_Bias	4463	0.1020	0.0666	0.0131	0.5000

**Table 2:** Baseline regression result based on difference-in-differences (DID) model.

	(1)	(2)	(3)	(4)	(5)	(6)
Variables	TFEE	TFEE	TFEE	TFEE	TFEE	TFEE
Treated × Time	0.0991 ***	0.0283 ***	0.0269 ***	0.0421 ***	0.0249 ***	0.0237 ***
	(0.008)	(0.010)	(0.004)	(0.009)	(0.009)	(0.004)
Pgdp				0.0757 ***	0.0421 ***	0.0317 ***
				(0.013)	(0.014)	(0.005)
Pdensity				0.0229 **	0.0163 *	0.0748 ***
				(0.010)	(0.010)	(0.011)
Indstru				−0.1324 ***	−0.0668 *	−0.0333 *
				(0.039)	(0.039)	(0.019)
Pollu				−0.0166 ***	−0.0104 **	−0.0136 ***
				(0.004)	(0.004)	(0.002)
Price				−0.0702 ***	−0.1725 ***	−0.1578 ***
				(0.022)	(0.043)	(0.023)
Regulation				0.0126	−0.0027	−0.0006
				(0.009)	(0.010)	(0.004)
Strpub				−0.0372 ***	−0.0598 ***	−0.0630 ***
				(0.013)	(0.014)	(0.006)
Fdi				0.0025	0.0022	0.0003
				(0.002)	(0.002)	(0.001)
_cons	0.1747 ***	0.1350 ***	0.1350 ***	0.1900	1.0132 ***	0.7402 ***
	(0.005)	(0.004)	(0.004)	(0.132)	(0.248)	(0.135)
Observations	4495	4495	4495	4495	4495	4495
Year Fixed Effects	No	Yes	Yes	No	Yes	Yes
City Fixed Effects	No	No	Yes	No	No	Yes
R-squared	0.1421	0.3380	0.3380	0.3447	0.3748	0.3811

Note: Robust Standard errors in parentheses; * *p* < 0.1, ** *p* < 0.05, *** *p* < 0.01.

**Table 3:** Parallel trend test.

	(1)	(2)	(3)
Variables	TFEE	TFEE	TFEE
D^−6^	0.0024	−0.0073	0.0047
	(0.008)	(0.007)	(0.008)
D^−5^	0.0037	−0.0043	0.0037
	(0.008)	(0.007)	(0.008)
D^−4^	0.0051	0.0001	0.0058
	(0.008)	(0.007)	(0.008)
D^−3^	0.0069	0.0012	0.0050
	(0.008)	(0.007)	(0.008)
D^−2^	0.0120	0.0004	0.0104
	(0.008)	(0.007)	(0.008)
D^0^	0.0254 ***	0.0202 ***	0.0262 ***
	(0.008)	(0.007)	(0.008)
D^1^	0.0364 ***	0.0362 ***	0.0366 ***
	(0.008)	(0.007)	(0.008)
D^2^	0.0515 ***	0.0595 ***	0.0499 ***
	(0.008)	(0.007)	(0.008)
D^3^	0.0285 ***	0.0367 ***	0.0276 ***
	(0.010)	(0.009)	(0.009)
D^4^	0.0226 **	0.0347 ***	0.0227 **
	(0.009)	(0.009)	(0.009)
D^5^	0.0266 ***	0.0252 ***	0.0262 ***
	(0.009)	(0.009)	(0.009)
D^6^	0.0262 ***	0.0309 ***	0.0242 ***
	(0.009)	(0.009)	(0.009)
Pgdp		0.0745 ***	0.0316 ***
		(0.004)	(0.005)
Pdensity		0.0952 ***	0.0752 ***
		(0.011)	(0.011)
Indstru		−0.0952 ***	−0.0185
		(0.018)	(0.019)
Pollu		−0.0202 ***	−0.0140 ***
		(0.002)	(0.002)
Price		−0.0656 ***	−0.1558 ***
		(0.015)	(0.023)
Regulation		0.0160 ***	−0.0012
		(0.004)	(0.004)
Strpub		−0.0307 ***	−0.0635 ***
		(0.006)	(0.006)
Fdi		−0.0005	−0.0008
		(0.001)	(0.001)
_cons	0.1345 ***	−0.2336 **	0.7366 ***
	(0.005)	(0.093)	(0.134)
Observations	4495	4495	4495
Year FE	Yes	No	Yes
City FE	No	Yes	Yes
R-squared	0.3433	0.3532	0.3871

Note: Robust Standard errors in parentheses; ** *p* < 0.05, *** *p* < 0.01.

**Table 4:** Robustness test.

	(1)	(2)	(3)
Variables	SFEE	Policy Exclusions	PSM-DID
Treated × Time	0.0828 ***	0.0161 ***	0.0154 ***
	(0.013)	(0.004)	(0.005)
Pgdp	0.1909 ***	0.0456 ***	0.0365 ***
	(0.016)	(0.009)	(0.006)
Pdensity	0.1903 ***	0.0531 ***	0.0443 ***
	(0.033)	(0.019)	(0.013)
Indstru	0.3017 ***	−0.2212 ***	−0.0727 ***
	(0.058)	(0.029)	(0.023)
Pollu	−0.0210 ***	−0.0076 ***	−0.0105 ***
	(0.006)	(0.002)	(0.003)
Price	0.0326	−0.1395 ***	−0.1793 ***
	(0.070)	(0.021)	(0.029)
Regulation	−0.0434 ***	0.0108 **	0.0072
	(0.013)	(0.005)	(0.005)
Strpub	−0.1350 ***	−0.0625 ***	−0.0651 ***
	(0.020)	(0.006)	(0.008)
Fdi	−0.0065 *	−0.0012	0.0011
	(0.004)	(0.001)	(0.002)
_cons	−0.7839 *	0.6930 ***	0.9661 ***
	(0.412)	(0.171)	(0.168)
Observations	4495	2809	3194
Year Fixed Effects	Yes	Yes	Yes
City Fixed Effects	Yes	Yes	Yes
R-squared	0.5549	0.3145	0.3652

Note: Robust Standard errors in parentheses; * *p* < 0.1, ** *p* < 0.05, *** *p* < 0.01.

**Table 5:** Robustness test: dynamic analysis.

	(1)	(2)	(3)	(4)	(5)	(6)
Variables	TFEE	TFEE	TFEE	TFEE	TFEE	TFEE
A	2009–2012	2008–2013	2007–2014	2006–2015	2005–2016	2004–2017
Treated_2010_ × Time_First_	0.0159 ***	0.0120 **	0.0121 **	0.0130 **	0.0160 ***	0.0148 ***
	(0.005)	(0.006)	(0.005)	(0.005)	(0.005)	(0.005)
Observations	1110	1332	1998	2220	2664	3108
R-squared	0.2426	0.2337	0.2583	0.2970	0.3272	0.3477
B	2010–2013	2009–2014	2008–2015	2007–2016	2006–2017	2016–2018
Treated_2012_ × Time_Second_	0.0179 ***	0.0120 *	0.0142 **	0.0178 ***	0.0223 ***	
	(0.007)	(0.007)	(0.007)	(0.007)	(0.007)	
Treated_2017_ × Time_Third_						0.0446 ***
						(0.017)
Observations	887	1238	1589	1935	2277	549
R-squared	0.3156	0.2545	0.2980	0.3505	0.3713	0.1831
Controls	Yes	Yes	Yes	Yes	Yes	Yes
Year Fixed Effects	Yes	Yes	Yes	Yes	Yes	Yes
City Fixed Effects	Yes	Yes	Yes	Yes	Yes	Yes

Note: Robust Standard errors in parentheses; * *p* < 0.1, ** *p* < 0.05, *** *p* < 0.01.

**Table 6:** Parallel trend test of three pilots.

	(1)	(2)	(3)
Variables	Treated_2010_	Treated_2012_	Treated_2017_
D^−6^	−0.0082	0.0014	0.0050
	(0.010)	(0.012)	(0.015)
D^−5^	−0.0086	0.0025	0.0063
	(0.010)	(0.012)	(0.015)
D^−4^	−0.0028	0.0073	0.0141
	(0.010)	(0.012)	(0.015)
D^−3^	0.0020	0.0033	0.0108
	(0.010)	(0.012)	(0.015)
D^−2^	0.0038	0.0037	0.0234
	(0.010)	(0.012)	(0.015)
D^0^	0.0098	0.0245 **	0.0649 ***
	(0.010)	(0.012)	(0.015)
D^1^	0.0201 **	0.0309 **	0.1004 ***
	(0.010)	(0.012)	(0.015)
D^2^	0.0267 ***	0.0251 **	0.1317 ***
	(0.010)	(0.012)	(0.015)
Pgdp	0.0644 ***	0.0784 ***	0.0353 ***
	(0.012)	(0.010)	(0.006)
Pdensity	0.0734 ***	0.0541 **	0.0351 ***
	(0.023)	(0.022)	(0.011)
Indstru	−0.2554 ***	−0.2388 ***	0.0232
	(0.034)	(0.028)	(0.022)
Pollu	−0.0077 ***	−0.0052 **	−0.0048 **
	(0.003)	(0.003)	(0.002)
Price	−0.1414 ***	−0.1031 ***	−0.0661 **
	(0.025)	(0.021)	(0.026)
Regulation	0.0161 ***	0.0190 ***	−0.0008
	(0.006)	(0.004)	(0.005)
Strpub	−0.0636 ***	−0.0442 ***	−0.0663 ***
	(0.008)	(0.006)	(0.007)
Fdi	−0.0008	−0.0018	−0.0027 *
	(0.002)	(0.001)	(0.001)
_cons	0.4605 **	0.1397	0.4309 ***
	(0.209)	(0.184)	(0.151)
Observations	2220	2195	3008
Year Fixed Effects	Yes	Yes	Yes
City Fixed Effects	Yes	Yes	Yes
R-squared	0.2844	0.3552	0.3974

Note: Robust Standard errors in parentheses; * *p* < 0.1, ** *p* < 0.05, *** *p* < 0.01.

**Table 7:** Heterogeneity test: city size.

	(1)	(2)	(3)	(4)	(5)
Variables	SMC	LC	II-LC	I-LC	ELC
Treated × Time	−0.0381 ***	0.0263 ***	−0.0041	0.0199 **	0.0519 ***
	(0.012)	(0.004)	(0.008)	(0.008)	(0.007)
Pgdp	0.0608 ***	0.0297 ***	0.0222 **	0.0503 ***	0.0303 ***
	(0.017)	(0.005)	(0.009)	(0.013)	(0.008)
Pdensity	0.1208 ***	0.0709 ***	0.1429 ***	0.1233 ***	0.0127
	(0.044)	(0.011)	(0.025)	(0.044)	(0.014)
Indstru	0.2473 ***	−0.0504 ***	0.0706 **	−0.0339	−0.1190 ***
	(0.054)	(0.020)	(0.033)	(0.033)	(0.035)
Pollu	−0.0166 ***	−0.0139 ***	−0.0054	0.0030	−0.0261 ***
	(0.004)	(0.002)	(0.004)	(0.004)	(0.003)
Price	−0.0651	−0.1649 ***	−0.0304	−0.1411 ***	−0.2145 ***
	(0.050)	(0.024)	(0.039)	(0.044)	(0.040)
Regulation	−0.0047	0.0025	0.0241 ***	−0.0195 **	0.0007
	(0.009)	(0.004)	(0.008)	(0.008)	(0.007)
Strpub	−0.0133	−0.0627 ***	−0.0604 ***	−0.0622 ***	−0.0623 ***
	(0.016)	(0.007)	(0.012)	(0.012)	(0.010)
Fdi	−0.0011	0.0011	−0.0004	−0.0023	0.0038 *
	(0.002)	(0.001)	(0.002)	(0.002)	(0.002)
_cons	−0.5945	0.8121 ***	−0.3211	0.0914	1.5313 ***
	(0.390)	(0.140)	(0.257)	(0.338)	(0.219)
Observations	166	4329	1235	1231	1863
Year Fixed Effects	Yes	Yes	Yes	Yes	Yes
City Fixed Effects	Yes	Yes	Yes	Yes	Yes
R-squared	0.7350	0.3793	0.3319	0.3661	0.4389

Note: Robust Standard errors in parentheses; * *p* < 0.1, ** *p* < 0.05, *** *p* < 0.01.

**Table 8:** Heterogeneity test: pollutant emission intensity and financing constraints.

	(1)	(2)	(3)	(4)
Variables	LPEI	HPEI	LFC	HFC
Treated × Time	0.0095 **	0.0274 ***	0.0074	0.0369 ***
	(0.004)	(0.007)	(0.005)	(0.010)
Pgdp	0.0385 ***	0.0194 *	0.0597 ***	0.0249 ***
	(0.005)	(0.011)	(0.007)	(0.008)
Pdensity	0.1098 ***	0.1835 ***	0.0864 ***	0.0465 ***
	(0.014)	(0.028)	(0.023)	(0.013)
Indstru	0.0013	−0.0340	−0.0221	0.0353
	(0.017)	(0.040)	(0.020)	(0.031)
Pollu	−0.0070 ***	−0.0283 ***	0.0014	−0.0176 ***
	(0.002)	(0.005)	(0.002)	(0.004)
Price	0.0364 *	−0.3033 ***	0.0063	−0.1595 ***
	(0.021)	(0.043)	(0.019)	(0.044)
Regulation	−0.0060	0.0109	0.0014	−0.0078
	(0.004)	(0.007)	(0.004)	(0.009)
Strpub	−0.0417 ***	−0.0640 ***	−0.0412 ***	−0.0834 ***
	(0.006)	(0.012)	(0.005)	(0.014)
Fdi	−0.0012	0.0048 *	−0.0015	0.0049 *
	(0.001)	(0.003)	(0.001)	(0.003)
_cons	−0.6255 ***	1.0288 ***	−0.5608 ***	1.0100 ***
	(0.144)	(0.277)	(0.170)	(0.230)
Observations	2228	2267	2127	2368
Year Fixed Effects	Yes	Yes	Yes	Yes
City Fixed Effects	Yes	Yes	Yes	Yes
R-squared	0.4200	0.4323	0.2810	0.3989

Note: Robust Standard errors in parentheses; * *p* < 0.1, ** *p* < 0.05, *** *p* < 0.01.

**Table 9:** Mediating effect test: technological transformation and technological innovation.

	(1)	(2)	(3)	(4)
Variables	Tech_trans	TFEE	Tech_Inno	TFEE
Treated × Time	0.1026 ***	0.0248 ***	0.1138 ***	0.0133 ***
	(0.026)	(0.004)	(0.015)	(0.004)
Tech_trans		0.0148 ***		
		(0.003)		
Tech_Inno				0.0903 ***
				(0.004)
Pgdp	0.8883 ***	0.0326 ***	−0.1192 ***	0.0507 ***
	(0.028)	(0.006)	(0.021)	(0.006)
Pdensity	0.5510 ***	0.0757 ***	0.0466	0.0781 ***
	(0.071)	(0.011)	(0.039)	(0.011)
Indstru	−0.5705 ***	−0.0444 **	−0.5751 ***	0.0039
	(0.116)	(0.020)	(0.069)	(0.019)
Pollu	−0.0399 ***	−0.0155 ***	−0.0727 ***	−0.0083 ***
	(0.011)	(0.002)	(0.007)	(0.002)
Price	−0.0835	−0.1605 ***	−1.0188 ***	−0.0684 ***
	(0.096)	(0.023)	(0.080)	(0.022)
Regulation	0.1429 ***	−0.0013	−0.0787 ***	0.0069 *
	(0.025)	(0.004)	(0.015)	(0.004)
Strpub	0.3688 ***	−0.0607 ***	−0.0705 ***	−0.0521 ***
	(0.037)	(0.007)	(0.022)	(0.006)
Fdi	0.0443 ***	−0.0002	−0.0055	0.0004
	(0.008)	(0.001)	(0.004)	(0.001)
_cons	−10.5854 ***	0.7438 ***	6.9415 ***	0.0148
	(0.593)	(0.141)	(0.481)	(0.136)
Observations	4408	4408	4479	4479
Year Fixed Effects	Yes	Yes	Yes	Yes
City Fixed Effects	Yes	Yes	Yes	Yes
R-squared	0.4821	0.3869	0.4056	0.4400
Mediation effect test results	Partial mediation effect		Partial mediation effect	
	$\frac{\theta_{1}\omega_{2}}{\alpha_{1}}=6.41\%$		$\frac{\theta_{1}\omega_{2}}{\alpha_{1}}=43.36\%$	

Note: Robust Standard errors in parentheses; * *p* < 0.1, ** *p* < 0.05, *** *p* < 0.01.6.1.2. Green technology innovation and green technology bias.

**Table 10:** Mediating effect test: green technology innovation and green technology bias.

	(1)	(2)	(3)	(4)
Variables	Green_Inno	TFEE	Green_Bias	TFEE
Treated × Time	0.0045 ***	0.0157 ***	0.0193 ***	0.0209 ***
	(0.001)	(0.004)	(0.003)	(0.004)
Green_Inno		1.6634 ***		
		(0.090)		
Green_Bias				0.1000 ***
				(0.021)
Pgdp	−0.0059 ***	0.0474 ***	−0.0258 ***	0.0407 ***
	(0.001)	(0.006)	(0.004)	(0.006)
Pdensity	0.0066 ***	0.0717 ***	0.0265 ***	0.0807 ***
	(0.002)	(0.011)	(0.008)	(0.011)
Indstru	−0.0261 ***	0.0119	−0.0457 ***	−0.0287
	(0.003)	(0.019)	(0.015)	(0.020)
Pollu	−0.0024 ***	−0.0114 ***	−0.0049 ***	−0.0145 ***
	(0.000)	(0.002)	(0.002)	(0.002)
Price	−0.0349 ***	−0.1018 ***	−0.0060	−0.1561 ***
	(0.004)	(0.022)	(0.017)	(0.023)
Regulation	−0.0021 ***	0.0044	0.0057 *	−0.0006
	(0.001)	(0.004)	(0.003)	(0.004)
Strpub	−0.0022 **	−0.0509 ***	−0.0127 ***	−0.0542 ***
	(0.001)	(0.006)	(0.005)	(0.006)
Fdi	−0.0003	0.0000	−0.0023 **	−0.0001
	(0.000)	(0.001)	(0.001)	(0.001)
_cons	0.2250 ***	0.2545 *	0.3372 ***	0.5740 ***
	(0.023)	(0.135)	(0.102)	(0.137)
Observations	4417	4417	4463	4463
Year Fixed Effects	Yes	Yes	Yes	Yes
City Fixed Effects	Yes	Yes	Yes	Yes
R-squared	0.4618	0.4381	0.1515	0.3947
Mediation effect test results	Partial mediation effect		Partial mediation effect	
	$\frac{\theta_{1}\omega_{2}}{\alpha_{1}}=31.58\%$		$\frac{\theta_{1}\omega_{2}}{\alpha_{1}}=8.14\%$	

Note: Robust Standard errors in parentheses; * *p* < 0.1, ** *p* < 0.05, *** *p* < 0.01.

**Table 11:** Parallel trend test of mediating variables.

	(1)	(2)	(3)	(4)
Variables	Tech_trans	Tech_Inno	Green_Inno	Green_Bias
D^−6^	−0.0220	−0.0387	−0.0011	−0.0040
	(0.043)	(0.031)	(0.002)	(0.006)
D^−5^	−0.1330	−0.0336	−0.0014	−0.0041
	(0.088)	(0.031)	(0.002)	(0.006)
D^−4^	0.0056	−0.0275	−0.0011	−0.0013
	(0.038)	(0.032)	(0.002)	(0.006)
D^−3^	0.1028	−0.0068	−0.0001	0.0022
	(0.088)	(0.031)	(0.002)	(0.006)
D^−2^	−0.0776	−0.0065	0.0015	0.0067
	(0.084)	(0.032)	(0.002)	(0.006)
D^0^	0.2183 ***	0.0229	0.0031 *	0.0060
	(0.074)	(0.032)	(0.002)	(0.006)
D^1^	0.1204 ***	0.0351	0.0032 *	0.0146 ***
	(0.039)	(0.031)	(0.002)	(0.006)
D^2^	0.1527 ***	0.0646 **	0.0049 **	0.0191 ***
	(0.038)	(0.032)	(0.002)	(0.007)
D^3^	0.2240 ***	0.1032 ***	0.0044 **	0.0200 ***
	(0.040)	(0.032)	(0.002)	(0.007)
D^4^	0.1921 ***	0.1213 ***	0.0051 **	0.0133 *
	(0.045)	(0.032)	(0.002)	(0.007)
D^5^	0.1815 ***	0.1191 ***	0.0041 **	0.0134 **
	(0.051)	(0.031)	(0.002)	(0.007)
D^6^	0.1427 ***	0.0991 ***	0.0026	0.0079
	(0.053)	(0.031)	(0.002)	(0.007)
Pgdp	0.8954 ***	−0.0837 ***	−0.0043 *	−0.0261 ***
	(0.055)	(0.022)	(0.002)	(0.004)
Pdensity	0.4999	−0.0048	0.0051	0.0271 ***
	(0.305)	(0.039)	(0.009)	(0.008)
Indstru	−0.6110 ***	−0.4785 ***	−0.0211 ***	−0.0401 ***
	(0.186)	(0.070)	(0.006)	(0.015)
Pollu	−0.0444 **	−0.0615 ***	−0.0021 ***	−0.0048 ***
	(0.018)	(0.007)	(0.001)	(0.002)
Price	−0.0595	−1.0548 ***	−0.0369 ***	−0.0088
	(0.118)	(0.080)	(0.008)	(0.017)
Regulation	0.1466 ***	−0.0652 ***	−0.0015	0.0054 *
	(0.039)	(0.015)	(0.001)	(0.003)
Strpub	0.3350 ***	−0.0528 **	−0.0018	−0.0128 ***
	(0.053)	(0.023)	(0.002)	(0.005)
Fdi	0.0364 ***	−0.0067	−0.0003	−0.0028 ***
	(0.012)	(0.004)	(0.000)	(0.001)
_cons	−10.1147 ***	6.8480 ***	0.2207 ***	0.3478 ***
	(1.671)	(0.482)	(0.073)	(0.102)
Observations	4408	4479	4417	4463
Year Fixed Effects	Yes	Yes	Yes	Yes
City Fixed Effects	Yes	Yes	Yes	Yes
R-squared	0.4935	0.3804	0.4419	0.1492

Note: Robust Standard errors in parentheses; * *p* < 0.1, ** *p* < 0.05, *** *p* < 0.01.

**Table 12:** Low-carbon city pilot, energy efficiency, and carbon emissions.

	(1)	(2)	(3)
Variables	CO_2_	TFEE	CO_2_
Treated × Time	−0.0177 ***	0.0237 ***	−0.0161 ***
	(0.006)	(0.004)	(0.006)
TFEE			−0.0705 ***
			(0.020)
Pgdp	0.0894 ***	0.0317 ***	0.0916 ***
	(0.007)	(0.005)	(0.007)
Pdensity	0.0637 ***	0.0748 ***	0.0690 ***
	(0.014)	(0.011)	(0.014)
Indstru	0.0977 ***	−0.0333 *	0.0954 ***
	(0.024)	(0.019)	(0.024)
Pollu	0.0233 ***	−0.0136 ***	0.0223 ***
	(0.003)	(0.002)	(0.003)
Price	0.1042 ***	−0.1578 ***	0.0930 ***
	(0.029)	(0.023)	(0.029)
Regulation	0.1061 ***	−0.0006	0.1060 ***
	(0.005)	(0.004)	(0.005)
Strpub	−0.0324 ***	−0.0630 ***	−0.0369 ***
	(0.008)	(0.006)	(0.008)
Fdi	−0.0022	0.0003	−0.0022
	(0.002)	(0.001)	(0.002)
_cons	0.6272 ***	0.7402 ***	0.6794 ***
	(0.173)	(0.135)	(0.174)
Observations	4495	4495	4495
Year Fixed Effects	Yes	Yes	Yes
City Fixed Effects	Yes	Yes	Yes
R-squared	0.9134	0.3811	0.9136
Mediation effect	Partial mediation effect		
test results	$\frac{\theta_{1}\omega_{2}}{\alpha_{1}}=9.44\%$		

Note: Robust Standard errors in parentheses; * *p* < 0.1, *** *p* < 0.01.

**Table 13:** Parallel Trend Test: carbon reduction.

	(1)	(2)
Variables	CO_2_	CO_2_
D^−6^	0.0067	0.0108
	(0.011)	(0.010)
D^−5^	0.0018	0.0091
	(0.011)	(0.010)
D^−4^	−0.0048	0.0019
	(0.011)	(0.010)
D^−3^	−0.0073	0.0029
	(0.011)	(0.010)
D^−2^	−0.0086	0.0057
	(0.011)	(0.010)
D^0^	−0.0424 ***	−0.0239 **
	(0.013)	(0.012)
D^1^	−0.0647 ***	−0.0499 ***
	(0.013)	(0.012)
D^2^	−0.0674 ***	−0.0555 ***
	(0.013)	(0.012)
D^3^	−0.0689 ***	−0.0606 ***
	(0.013)	(0.012)
D^4^	−0.0659 ***	−0.0505 ***
	(0.013)	(0.012)
D^5^	−0.0695 ***	−0.0606 ***
	(0.016)	(0.015)
D^6^	−0.0692 ***	−0.0645 ***
	(0.016)	(0.014)
Pgdp		0.0880 ***
		(0.007)
Pdensity		0.0639 ***
		(0.014)
Indstru		0.1171 ***
		(0.024)
Pollu		0.0231 ***
		(0.003)
Price		0.1017 ***
		(0.029)
Regulation		0.1052 ***
		(0.005)
Strpub		−0.0319 ***
		(0.008)
Fdi		−0.0032 **
		(0.002)
_cons	2.3112 ***	0.6443 ***
	(0.006)	(0.171)
Observations	4495	4495
Year Fixed Effects	Yes	Yes
City Fixed Effects	Yes	Yes
R-squared	0.8941	0.9151

Note: Robust Standard errors in parentheses; ** *p* < 0.05, *** *p* < 0.01.

**Table 14:** Energy rebound effect: all cities, pilot cities, and nonpilot cities.

Year	All Cities	Nonpilot Cities	Pilot Cities	
			Post>=2010	Post>=2012
2004	0.9829	0.9542	0.9264	0.9601
2005	0.6413	0.6206	0.7072	0.6905
2006	0.7492	0.7320	0.7999	0.6344
2007	0.9296	0.8719	1.0917	0.9112
2008	0.7581	0.7238	0.8488	0.6979
2009	0.7261	0.7453	0.6779	0.9690
2010	0.8281	0.7962	0.9213	0.7986
2011	0.7160	0.6793	0.8086	0.7594
2012	1.0330	1.0102	1.0887	1.0625
2013	0.8764	0.8427	0.9474	1.0215
2014	0.5362	0.5134	0.5957	0.9089
2015	1.2456	1.2439	1.2496	1.6656
2016	0.8358	0.8005	0.9360	0.9814
2017	1.0661	1.0156	1.1896	1.2144
Mean	0.8518	0.8250	0.9135	0.9482

Note: When calculating the energy rebound effect value, the following two situations need to be excluded: (1) years when energy intensity increases; (2) years when energy intensity decreases, but the contribution rate of energy efficiency to output growth is negative.

## Data Availability

The data presented in this study are available on request from the authors.
